# Mutations in Growth-Related Genes Induced by EMS Treatment in Scallops

**DOI:** 10.3389/fgene.2022.879844

**Published:** 2022-04-26

**Authors:** Caihui Wang, Bo Liu, Min Chen, Junhao Ning, Xia Lu, Chunde Wang

**Affiliations:** ^1^ School of Marine Science and Engineering, Qingdao Agricultural University, Qingdao, China; ^2^ Yantai Institute of Coastal Zone Research, Chinese Academy of Sciences, Yantai, China

**Keywords:** EMS, mutagen, scallop, genome sequencing, SNP, INDEL, gene

## Abstract

**Background:** The goal of genetic breeding is to select variants with mutations that are related to expected traits, such as fast growth. Artificial induction has been widely used to obtain strains with more mutations for further selection. Ethylmethylsulfone (EMS) is one of the most commonly used chemical mutagens in plant and microorganism breeding. However, the application of EMS mutagenesis in shellfish has not been reported. The aim of this study is to evaluate the potential use of EMS as a mutagen in scallop breeding, especially in characterization of mutations in growth-related genes.

**Results:** Our results indicated that hatching of about 50% of fertilized eggs was blocked by treatment with 20 mM EMS for 3 h and the resulted larvae developed normally into adult stages. We then evaluated the mutagenic effects of EMS by sequencing the genomes of 4 adult scallops from the control group and 12 from the treatment group at 8 months after fertilization. On average, after removing shared types of mutations, there were 1,151,380 ± 258,188 SNPs (Single Nucleotide Polymorphisms) and 229,256 ± 51,714 InDels (insertion-deletion) in each animal in the EMS treatment group, while there were only134841 ± 10,115 SNPs and 42,605 ± 5,136 InDels in the control group. The average mutation rate in the genome of the EMS treatment group (0.0137 ± 0.0013%) was about 9 times that of the control group (0.0015 ± 0.0002%). GO (Gene Ontology) annotation and KEGG (Kyoto Encyclopedia of Genes and Genomes) enrichment analyses revealed that mutations induced by EMS occurred evenly in most biological processes, cellular components and functions, as well in most pathways. However, significant lower percentage of mutations were found in the exonic region, in non-synonymous or Stopgain/Stoploss SNPs and in coding domains, suggesting apparent DNA repair or selection during grow-out stage. Analyses of the growth-related genes with mutations indicated that mutations in MFS (Major Facilitator Superfamily) and Tubulin were only found in the large-sized group (Five largest scallops: Treated-1, Treated-2, Treated-3, Treated-4, and Treated-5) and Homeobox and Socs (Suppressor of cytokine signaling) only in the small group (Two smallest scallops: Treated-11 and Treated-12). These results suggested that these genes may be involved in the regulation of growth in these animals, although further verification is certainly warranted.

**Conclusion:** Treatment of fertilized eggs with 20 mM EMS for 3 h induced 9 times more mutations in scallop genomes. We found that mutations in MFS and Tubulin may be related to fast growth in the large-sized group and those mutations in Homeobox and SOCs may be involved in the slow growth in the small-sized scallops. EMS can be used to accelerate selection of economically important traits in molluscs.

## Background

The purpose of genetic breeding in aquaculture is to select variants with superior economically important traits, such as rapid growth, disease resistance and high survival. No matter which breeding method is used, successful selection depends on the availability of mutations in the base population. Mutations are the primary agents of long-term evolution. Genetic variation and naturally occurring mutations play an important role in evolution (Hou et al., 2019). Although naturally occurring mutations represent a major force driving evolution, the occurrence rate of spontaneous mutation is extremely low (Lian et al., 2020). Studies have shown that the natural mutation rate of specific loci in fish is generally lower than 1.0 × 10^–6^ (Kuroyanagi et al., 2013). The effective way to obtain a high mutation rate is by artificial induction. Artificially induced genetic variations represent important supplementary variation in plant breeding programs complementary to sources from natural origins (Lu et al., 2016). In recent years, artificial mutagenesis has been widely used in animal and plant breeding as well as in microbe breeding. For example, application of physical and chemical mutagenesis has led to great progress in the selection of algal strains with increased lipid production (Ornuma et al., 2018).

Physical and chemical approaches are the most commonly used artificial induction methods in the construction of plant mutant library (Chen et al., 2018). Compared with physical mutagenesis, chemical mutagenesis is more cost-effective and does not require complicated equipment. Ethylmethylsulfone (EMS) is one of the most commonly used chemical mutagens in breeding of plants such as wheat and Maize (Tran et al., 2020; Xiong et al., 2020). By alkylation of guanine, EMS tends to induce more point mutations from G:C to T:A (Till et al., 2007; Sikora et al., 2011). EMS has been used in the selection of new strains with increased lipid productivity in *Chlorella sp.* (Ornuma et al., 2018) and also new strain with elevated tolerance to salt in wheats (Johanna et al., 2020). In recent years, EMS chemical mutagenesis has also begun to be applied in animals such as *Drosophila* (Lee et al., 2016) and nematodes (Song et al., 2014). However, EMS has not been used in genetic breeding of molluscs.

The bay scallop *Argopecten irradians* is a commercially important bivalve widely cultured in northern China (Ning et al., 2018). The cultured population of the bay scallops originated from only a small number of brood stocks survived from the last attempt of 3 consecutive introductions in early 1980s (Zhang et al., 1986). Efforts have been devoted to improve the stocks through re-introduction of more brood stocks, hybridization and selective breeding. Selective breeding has been proven to be an effective approach for selection of variants with superior traits, mainly from populations with naturally occurring mutations (Wang et al., 2018). However, selective breeding based on naturally occurred mutations is limited by decreasing genetic diversity caused by continuous inbreeding and thus lack of selectable mutants (Zhao et al., 2019). Therefore, it is necessary to broaden the genetic diversity of the bay scallops by artificial induction. In the present study, we aimed to evaluate the feasibility of using EMS as a mutagen in the genetic breeding of scallops and characterize the mutations in growth-related genes.

## Materials and Methods

### Animals

The animals used in this study were ‘Bohai Red’ scallops, a new bay scallop strain selected from the hybrids between the bay scallop and the Peruvian scallop, which is widely cultured in northern China (Wang et al., 2017). The scallops were cultured in a scallop farm located in Yangma Island, Mouping, Shandong. They were conditioned to mature in the scallop hatchery of Yantai Spring-Sea AquaSeed Co., Ltd. located in Laizhou, Shandong.

### Determination of 50% Lethal Concentration of EMS Treatment

When the scallops became mature, they were induced to spawn individually in beakers of 1 L by exposing them to air for 30 min followed by a temperature shock from 18 to 23°C. The spawning scallops were watched carefully to collect eggs and sperm separately. Eggs or sperm from different scallops were pooled together. Then sperm were mixed with eggs to obtain fertilized eggs for subsequent experiments.

After fertilization, fertilized eggs were divided into six groups and a certain amount of EMS stock solution was added to the fertilized egg suspension so that the concentrations of EMS in the containers were 0, 10, 20, 30, 40, 50, and 60 mM. Three replicates were set for each group. EMS stock solution was purchased from Sigma (Ronkonkoma, United States). The fertilized eggs were exposed to EMS solutions of different concentrations for 3 h before they were filtered and washed with fresh seawater and kept in water bath for hatching at 23°C. The fertilized eggs were stirred every half hour.

Twenty-eight hours after fertilization, three 1 ml samples were taken from each container and observed under a microscope. Hatching rates, the percentages of D-formed larvae in total fertilized eggs, were determined for each container. The 50% lethal concentration of EMS was determined based on the hatching rates of fertilized eggs exposed to different concentrations of EMS.

### Animal Rearing and Sampling

The shell height and shell length of the larvae in each EMS concentration group were measured on Day 3, 6, 9, and 10 after fertilization to examine the effects of EMS on larval growth. Five 1 ml samples were randomly taken from each container for measurements. Ten days after fertilization, when over 50% of the larvae developed eyespots, the larvae were set on plastic collectors and allowed for metamorphosis. After metamorphosis, the set juveniles were taken first to the ponds and then to the open sea for nursery. When the juveniles reached 2 cm in shell height in late Aug., they were dispersed into lantern nets for grow-out.

About 8 months after fertilization, about 20,000 scallops were harvested and screened for individuals with apparent phenotypic mutation. As large phenotypic variations existed in shell height, shell length and shell width, we used the sum of shell height, shell length and shell width to select the largest and smallest individuals. As a result, 5 largest (Treated-1, Treated-2, Treated-3, Treated-4, and Treated-5), 2 smallest scallops (Treated-11 and Treated-12) and 5 normal-sized (Treated-6, Treated-7, Treated-8, Treated-9, and Treated-10) were selected from the two ends and middle of the normal distribution of individual shell lengths for whole genomic resequencing. Four scallops from the untreated control group were also randomly selected for whole genome sequencing. The sizes of the sequenced scallops were given in Table 1.

**TABLE 1:** Sizes of the sequenced individuals in the control and treatment group.

Groups	Sample ID	Length (mm)	Height (mm)	Width (mm)	Sum
Control group	Control_1	66	65	31	162
	Control_2	65	64	31	160
	Control_3	65	63.5	31	159.5
	Control_4	64	63	30	157
Large-sized treatment group	Treated_1	79.5	77	31	187.5
	Treated_2	77	66	32	175
	Treated_3	77	76	32	185
	Treated_4	77	76	33	186
	Treated_5	77	77	31	185
Normal-sized treatment group	Treated_6	66	64	31	161
	Treated_7	65	65.5	29	159.5
	Treated_8	64	51.5	29	144.5
	Treated_9	63	51	29	143
	Treated_10	59	58	28	145
Small-sized treatment group	Treated_11	51	49	28	128
	Treated_12	50	49.5	28.5	128

### Whole Genome Sequencing

After harvest, the scallops were dissected to collect their adductor muscles. DNA was extracted from each adductor muscle sample. And the qualified DNA was randomly broken into 350bp fragments by Covaris crusher, and NDM607-01 was used to build the database. The whole library was prepared by terminal repair, ployA tail addition, sequencing connector addition, purification and PCR amplification. After the library construction was completed, Qubit2.0 was used for preliminary quantification, and the library was diluted to 1 ng/μl. Agilent 2100 was then used to test the insert size of the library, and QPCR was used to accurately quantify the effective concentration of the library (effective concentration of the library >2 nM). And sequenced using paired-end Illumina platform.

### Evaluation of EMS-Induced Mutations

We aligned the raw data to the Bay scallop reference genome (BioSample accessions: SAMN08322131; Liu et al., 2020 using the mem algorithm in BWA (Burrows-Wheeler Alignment) (Li and Durbin, 2009). We then used Samtools to convert the file format and sort the subsequent alignment sequences (Li et al., 2009). Subsequently, GATK (The Genome Analysis Toolkit) was used to remove repeat sequences and to detect SNPs and InDels. SNPs and InDels specific to each group were obtained by removing shared mutations identified by comparing their mutation information (including CHROM, POS, REF, ALT, and GENO) in both the control and treatment group. Then we annotated each variation with annovar to obtain information for further identification of synonymous and nonsynonymous mutations. Finally, SNPs/InDels were annotated to the reference genome using the annovar package (Alexander et al., 2009; Vilella et al., 2009; Wang et al., 2010; Danecek et al., 2011; Yang et al., 2011; Schiffels and Durbin, 2014; Zhang et al., 2018). For GO and KEGG enrichment analysis, the final results were obtained by performing enrichment analysis using hypergeometric test, followed by FDR (False Discovery Rate) correction of the *p*-values.

### Screening of Mutations in Growth-Related Genes

We compared the genes with nonsynonymous SNP sites in the large- and small-sized individuals against controls and normal-sized individuals to find the mutated genes that appeared in only large- or small-sized individuals. From these mutated genes, we then searched for genes that are potentially related to growth.

### Statistics and Data Analyses

Statistical analyses of the data were performed using the SPSS (Statistical Product and Service Solutions) statistical package (SPSS Science, Chicago, Illinois). Briefly, the raw data on size parameters in different treated group and hatching rate were first tested for normality of distribution using the Kolmogorov-Smirnov and Shapiro-Wilk tests, and for homogeneity of the variance using the Levene tests. If the raw data failed these tests, square root or natural logarithm transformations were then performed. When both the normality and homogeneity were met, the differences in growth and hatching rate were analyzed with one-way analysis of variance (ANOVA) followed by multiple comparison tests (Tukey’s test) (Wang et al., 2010).

## Results

### Determination of the 50% Lethal Concentration of EMS Treatment

**FIGURE 1:**
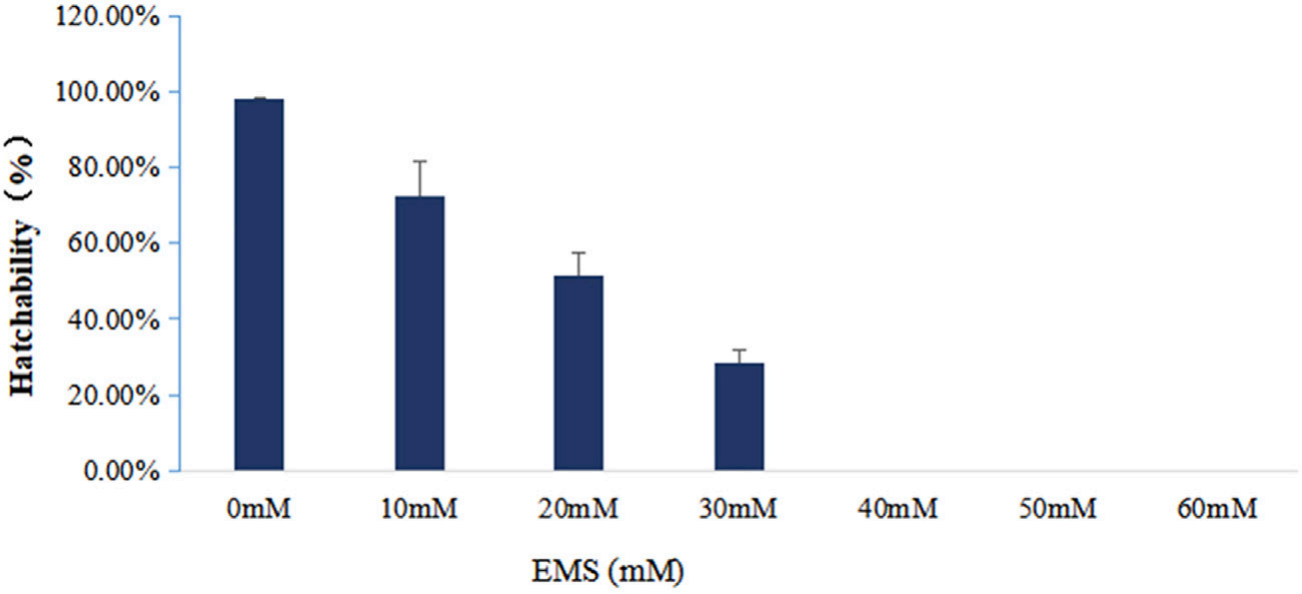
Hatching rate of fertilized eggs treated with different concentrations of EMS.

**FIGURE 2:**
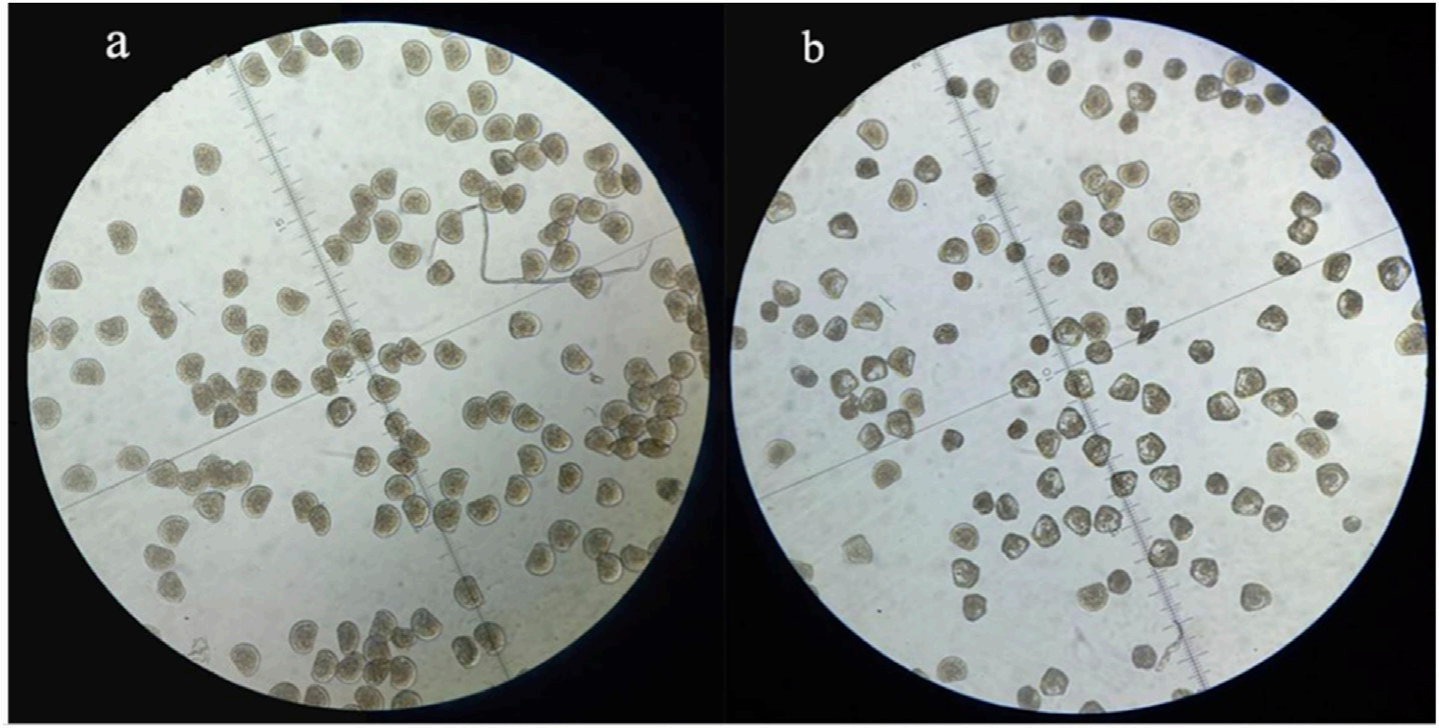
D-formed larvae at 28 h after fertilization in the control **(A)** and the EMS treatment group **(B)**.

To figure out the appropriate dose for EMS treatment, we first determined the 50% lethal concentration of EMS by examining the hatching rates of fertilized eggs treated with different concentration of EMS. Our results showed that EMS inhibited hatching rate in a dose-dependent manner. In the treatment groups, some fertilized eggs either developed into abnormal embryos or ceased to develop at early stages (Figure 1). Compared with the control group, about 50% of the fertilized eggs was blocked from hatching by 20 mM EMS (Figure 2). Therefore, we chose 20 mM EMS in subsequent experiments.

### Effects of EMS Treatment on Larval Growth

**FIGURE 3:**
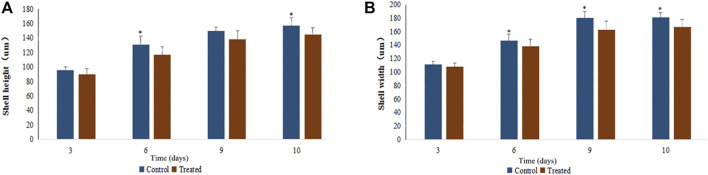
Effects of EMS on growth in shell height **(A)** and shell width **(B)** of scallop larvae.

EMS exhibited slightly inhibitory effects on larval growth. As can be seen in Figure 3, the growth curve of shell height and shell length of the treatment group (20 mM EMS) were significantly different from that of the control group during larval stage (*p* < 0.05, ANOVA). Shell height on day 6 and day 10 and shell length on Day 6, 9, and 10 in the control group were significantly larger than those of the EMS treatment group (*p* < 0.05, *t*-test).

### Genomic Analyses of EMS-Induced Mutations

To identify the mutations induced by EMS that may have contributed to important traits such as growth, we re-sequenced the genomes of the largest and smallest individuals from the treatment group, as well as those of the control group. In total, we obtained 156.9 Gb clean data from these 16 animals. The Q30 values of all samples were higher than 80%, indicating that the data were of low error rate and high quality (Table 2). The average mapping rate to the reference genome sequence was 99.54% with an average sequencing depth of 11.19× (Table 3).

**TABLE 2:** Genome sequencing data.

Sample ID	Clean Reads pairs	Clean base (bp)	Length	Q20 (%)	Q30 (%)	GC (%)
Control_1	72,397,388	10,730,101,234	148.34; 148.08	96.8; 97.1	89.0; 89.6	35.0; 34.9
Control_2	86,746,906	12,841,328,031	148.15; 147.92	96.4; 96.9	88.1; 89.0	35.5; 35.3
Control_3	78,575,284	11,637,694,182	148.29; 147.92	96.8; 96.9	88.9; 89.2	35.1; 35.0
Control_4	80,070,256	11,846,376,980	148.14; 147.76	96.5; 96.7	88.2; 88.6	36.1; 36.0
Treated_1	67,832,690	10,041,975,926	148.2; 147.88	97.3; 97.6	90.2; 91.0	35.9; 35.9
Treated_2	64,159,190	9,470,920,591	148.25; 146.98	96.7; 96.3	88.7; 87.3	35.4; 35.4
Treated_3	63,990,224	9,454,811,079	147.95; 147.56	96.3; 96.5	87.3; 87.6	35.1; 35.1
Treated_4	65,044,560	9,615,563,726	148.02; 147.64	96.4; 96.6	87.5; 87.7	34.9; 34.8
Treated_5	67,712,528	10,006,244,700	148.06; 147.49	96.5; 96.4	87.8; 87.4	35.7; 35.7
Treated_6	65,341,510	9,667,122,876	148.06; 147.84	96.5; 96.8	87.9; 88.3	35.7; 35.7
Treated_7	66,519,728	9,826,753,951	148.07; 147.38	96.3; 96.3	87.3; 86.9	34.8; 34.7
Treated_8	66,587,084	9,863,157,597	148.17; 148.08	96.7; 97.0	88.2; 89.1	34.9; 34.8
Treated_9	78,747,896	11,665,293,124	148.25; 148.02	96.8; 97.1	89.0; 89.6	35.2; 35.1
Treated_10	64,408,982	9,530,201,612	148.16; 147.77	96.7; 96.9	88.8; 89.2	34.8; 34.7
Treated_11	68,486,448	10,122,154,287	148.18; 147.42	96.5; 96.3	88.2; 87.5	35.7; 35.7
Treated_12	74,752,348	11,073,164,954	148.29; 147.97	96.7; 96.9	88.7; 89.1	34.9; 34.7

**TABLE 3:** Summary of assembly results.

Sample	Average_sequencing_depth (x)	Coverage (%)	Mapped (%)	Properly mapped (%)	Singletons mapped (%)
Control_1	11.49	84.29	99.59	91.16	0.15
Control_2	13.36	84.71	99.61	90.77	0.15
Control_3	12.58	84.55	99.6	91.11	0.15
Control_4	12.51	84.69	99.59	90.51	0.15
Treated_1	10.65	83.82	99.67	92.19	0.13
Treated_2	10.14	84.00	99.24	90.11	0.17
Treated_3	10.22	82.57	99.49	90.62	0.17
Treated_4	10.52	83.87	99.55	90.93	0.17
Treated_5	10.66	84.24	99.55	90.81	0.16
Treated_6	10.29	84.03	99.61	91.41	0.15
Treated_7	10.66	82.87	99.54	90.68	0.18
Treated_8	10.81	83.96	99.54	91.15	0.15
Treated_9	12.48	84.60	99.61	90.47	0.14
Treated_10	10.52	82.92	99.56	90.60	0.17
Treated_11	10.85	84.53	99.61	90.80	0.15
Treated_12	12.12	84.45	99.52	90.97	0.15

#### SNPs

The average number of SNPs in the control group (5,866,344 ± 95,080) is not significantly different from that of the EMS treatment group (5,626,416 ± 297,062) (Table 4). However, after removing the shared SNPs with the control group, significantly more SNPs were found in the EMS treatment group than the control group (*p* < 0.05, *t*-test). As can be seen from Table 4, the number of SNPs in the control group was 134,841 ± 10,115 while that in the EMS treatment group was 1,151,380 ± 258,188. The SNPs are mainly located in intergenic regions, followed by intronic regions, and the rest were located in upstream and exon region. The Ts/Tv (Transitions/Transversions) value in the EMS treatment group was significantly higher than that in the control group (*p < 0.05*) (Table 5). (Transitions means a transformation between nucleotides of the same type; Transversions means a transformation between nucleotides of the different type).

**TABLE 4:** SNP information in the control and EMS treatment group after calling.

Sample ID	SNP num	Transition	Transversion	Ts/Tv
Control_1	5,734,870	2,994,839	2,649,234	1.13
Control_2	5,962,010	3,114,468	2,754,571	1.13
Control_3	5,888,822	3,075,869	2,721,273	1.13
Control_4	5,879,675	3,075,363	2,711,445	1.13
Treated_1	5,595,911	2,927,478	2,576,977	1.14
Treated_2	5,655,161	2,958,453	2,606,812	1.13
Treated_3	5,174,423	2,699,803	2,377,189	1.14
Treated_4	5,769,920	3,016,864	2,662,803	1.13
Treated_5	5,658,279	2,961,110	2,606,843	1.14
Treated_6	5,704,352	2,985,503	2,628,324	1.14
Treated_7	5,404,289	2,820,629	2,488,661	1.13
Treated_8	5,635,551	2,942,406	2,602,225	1.13
Treated_9	6,012,300	3,141,694	2,778,385	1.13
Treated_10	5,037,014	2,622,721	2,320,483	1.13
Treated_11	5,913,470	3,095,125	2,728,073	1.13
Treated_12	5,956,325	3,110,318	2,754,478	1.13

**TABLE 5:** SNP number in the control and EMS treated group after removing shared types with controls.

Sample ID	SNP Number	Transition	Transversion	Ts/Tv	Exonic	Intronic	Mutation rate (%)
Control_1	144,157	74,612	69,545	1.07	5,221	55,337	0.001343
Control_2	133,641	69,136	64,505	1.07	5,076	47,083	0.001041
Control_3	140,421	72,744	67,677	1.07	5,093	52,157	0.001207
Control_4	121,144	63,020	58,124	1.08	4,217	45,983	0.001023
Treated_1	919,124	486,397	432,727	1.12	33,221	343,986	0.009153
Treated_2	899,577	474,911	424,666	1.12	31,786	338,180	0.009498
Treated_3	1,259,663	665,325	594,338	1.12	44,543	480,891	0.013323
Treated_4	836,603	441,775	394,828	1.12	28,400	317,373	0.008701
Treated_5	884,410	467,731	416,679	1.12	31,431	336,862	0.008839
Treated_6	844,165	447,477	396,688	1.13	31,064	322,816	0.008732
Treated_7	1,301,091	686,338	614,753	1.12	43,564	499,773	0.013240
Treated_8	1,283,904	676,586	607,318	1.11	43,240	484,351	0.013017
Treated_9	1,479,438	779,953	699,485	1.12	49,384	557,013	0.012682
Treated_10	1,188,795	625,965	562,830	1.11	40,539	451,110	0.012474
Treated_11	1,452,761	768,189	684,572	1.12	51,409	553,680	0.014352
Treated_12	1,467,033	772,501	694,532	1.11	48,801	547,507	0.013249

The results also showed that the average number of non-synonymous SNP in the treatment group (17,020 ± 3,449) were much higher than those in the controls (2471 ± 275). The average number of Stopgain/Stoploss SNPs in the treatment group (358 ± 60) was also higher than that of the control group (64 ± 9) (Table 6).

**TABLE 6:** SNP mutation types in the control and EMS treatment groups.

Sample ID	Synonymous SNP	Non-synonymous SNP	Stopgain	Stoploss
Control_1	2453	2664	62	7
Control_2	2343	2604	68	6
Control_3	2461	2551	52	9
Control_4	2057	2065	49	4
Treated_1	18,232	14,477	287	33
Treated_2	17,661	13,667	278	31
Treated_3	24,730	19,120	363	36
Treated_4	15,773	12,174	235	24
Treated_5	17,551	13,420	289	25
Treated_6	17,308	13,317	248	27
Treated_7	24,464	18,451	365	46
Treated_8	24,100	18,551	344	43
Treated_9	27,652	21,062	367	50
Treated_10	22,710	17,300	310	52
Treated_11	29,038	21,696	366	49
Treated_12	27,137	21,004	375	53

**FIGURE 4:**
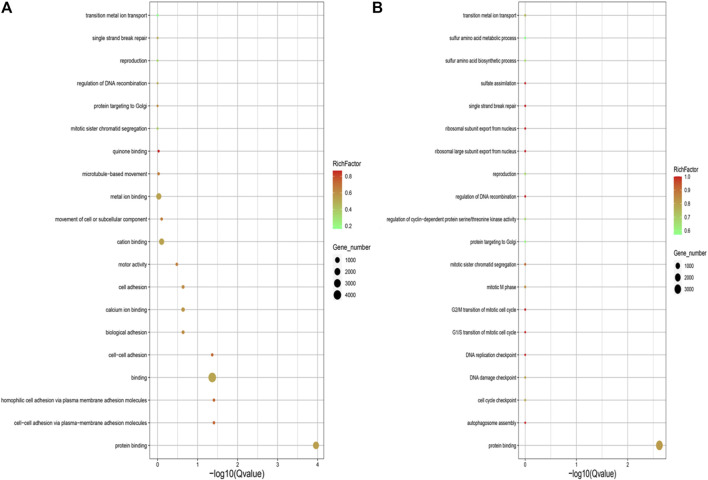
**(A)** GO annotation analysis of SNPs located in upstream and exon region in the control group. **(B)** GO annotation analysis of SNPs located in upstream and exon region in the EMS treatment group.

GO enrichment analysis for the genes with SNPs located in the upstream and exon regions revealed that genes were significantly enriched in the GO terms of protein binding, cell-cell adhesion via plasma-membrane adhesion molecules, homophilic-cell adhesion via plasma-membrane adhesion molecules, binding and cell-cell adhesion in the EMS treatment group, but the genes were only enriched in GO term of protein binding in the control group (Figure 4).

**FIGURE 5:**
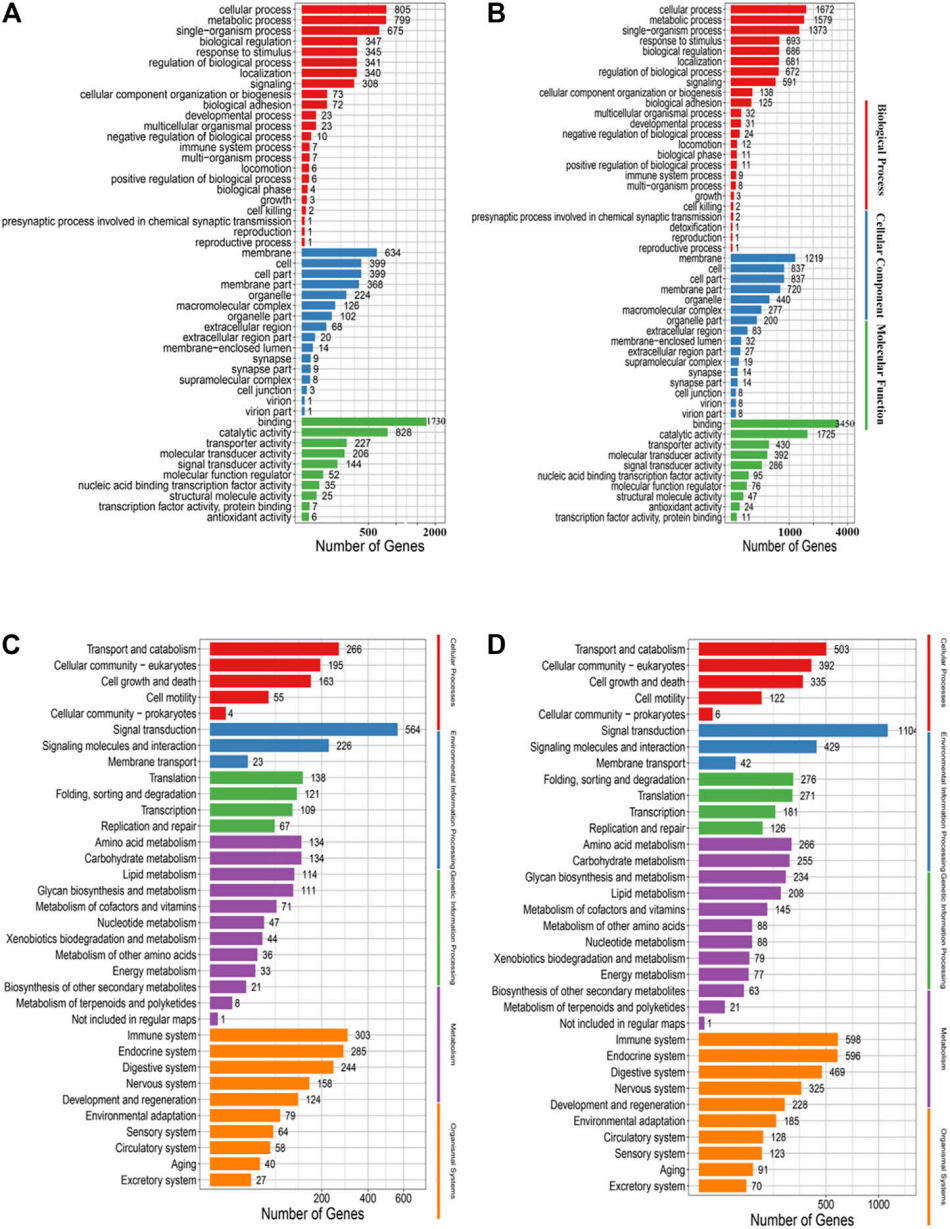
**(A)** GO annotation analysis of nonsynonymous SNPs in the control group. **(B)** GO annotation analysis of nonsynonymous SNPs in the treatment group. **(C)** KEGG enrichment analysis of nonsynonymous SNPs in the control group. **(D)** KEGG enrichment analysis of nonsynonymous SNPs in the treatment group.

GO and KEGG analyses of the genes with mutations showed that there were no distinct distribution for the GO terms that were classified into the biological processes, cellular components and molecular functions between the EMS-treated group and control group, as well as for the KEGG pathways (Figure 5).

#### InDels

The average numbers of InDels in the genome and in the CDs of the control group (1,548,571 ± 28,720 in the genome and 9,016 ± 310 in the CDs) was also not significantly different from that of the EMS treatment group (1,462,511 ± 83,433 in the genome and 8,518 ± 552 in the CDs) (Table 7). After removing shared InDels in the controls, the average numbers of InDels in the genome and in the CDs of the EMS treatment group (229,256 ± 51,714 in the genome and 1798 ± 302 in the CDs) were much higher than those in the control group (42,605 ± 5,136 in the genome and 640 ± 15 in the CDs) (Table 8).

**TABLE 7:** InDels in the control and EMS treatment group after calling.

Sample	Genome total	Genome insertion	Genome deletion	CDS total	CDS insertion	CDS deletion
Control_1	1,511,400	681,992	829,408	8,606	3,515	5,091
Control_2	1,580,568	713,062	867,506	9,233	3,773	5,460
Control_3	1,556,430	701,814	854,616	8,946	3,658	5,288
Control_4	1,545,887	696,134	849,753	9,278	3,786	5,492
Treated_1	1,424,749	636,777	787,972	9,113	3,679	5,434
Treated_2	1,457,488	657,042	800,446	8,424	3,439	4,985
Treated_3	1,341,891	607,142	734,749	7,787	3,100	4,687
Treated_4	1,500,738	676,211	824,527	8,239	3,268	4,971
Treated_5	1,465,430	660,129	805,301	8,808	3,582	5,226
Treated_6	1,466,508	659,425	807,083	8,789	3,627	5,162
Treated_7	1,412,924	639,855	773,069	7,794	3,170	4,624
Treated_8	1,468,654	661,746	806,908	8,444	3,403	5,041
Treated_9	1,589,485	716,156	873,329	9,387	3,718	5,669
Treated_10	1,312,194	593,108	719,086	7,694	3,144	4,550
Treated_11	1,535,662	692,346	843,316	8,785	3,588	5,197
Treated_12	1,574,406	709,673	864,733	8,958	3,599	5,359

**TABLE 8:** InDels in the control and EMS treated group after removing shared types with controls.

Sample	Genome total	Genome insertion	Genome deletion	CDS total	CDS insertion	CDS deletion
Control_1	47,081	21,178	25,903	658	263	395
Control_2	38,755	17,315	21,440	634	236	398
Control_3	37,598	18,524	19,074	623	225	356
Control_4	46,987	17,568	29,419	645	261	387
Treated_1	167,843	76,623	101,422	1867	721	1,146
Treated_2	169,398	76,465	103,672	1,501	589	912
Treated_3	175,975	107,611	142,501	1913	668	1,245
Treated_4	178,045	73,190	96,208	1,260	432	828
Treated_5	180,137	112,309	147,840	1,525	589	936
Treated_6	238,232	76,062	99,913	1,518	599	919
Treated_7	250,112	72,360	95,483	1771	662	1,109
Treated_8	253,471	129,826	168,342	1847	664	1,183
Treated_9	260,149	109,500	143,971	2288	843	1,445
Treated_10	284,295	123,192	161,103	1837	696	1,141
Treated_11	295,252	128,607	166,645	2096	782	1,314
Treated_12	298,168	102,954	135,278	2151	777	1,347

**FIGURE 6:**
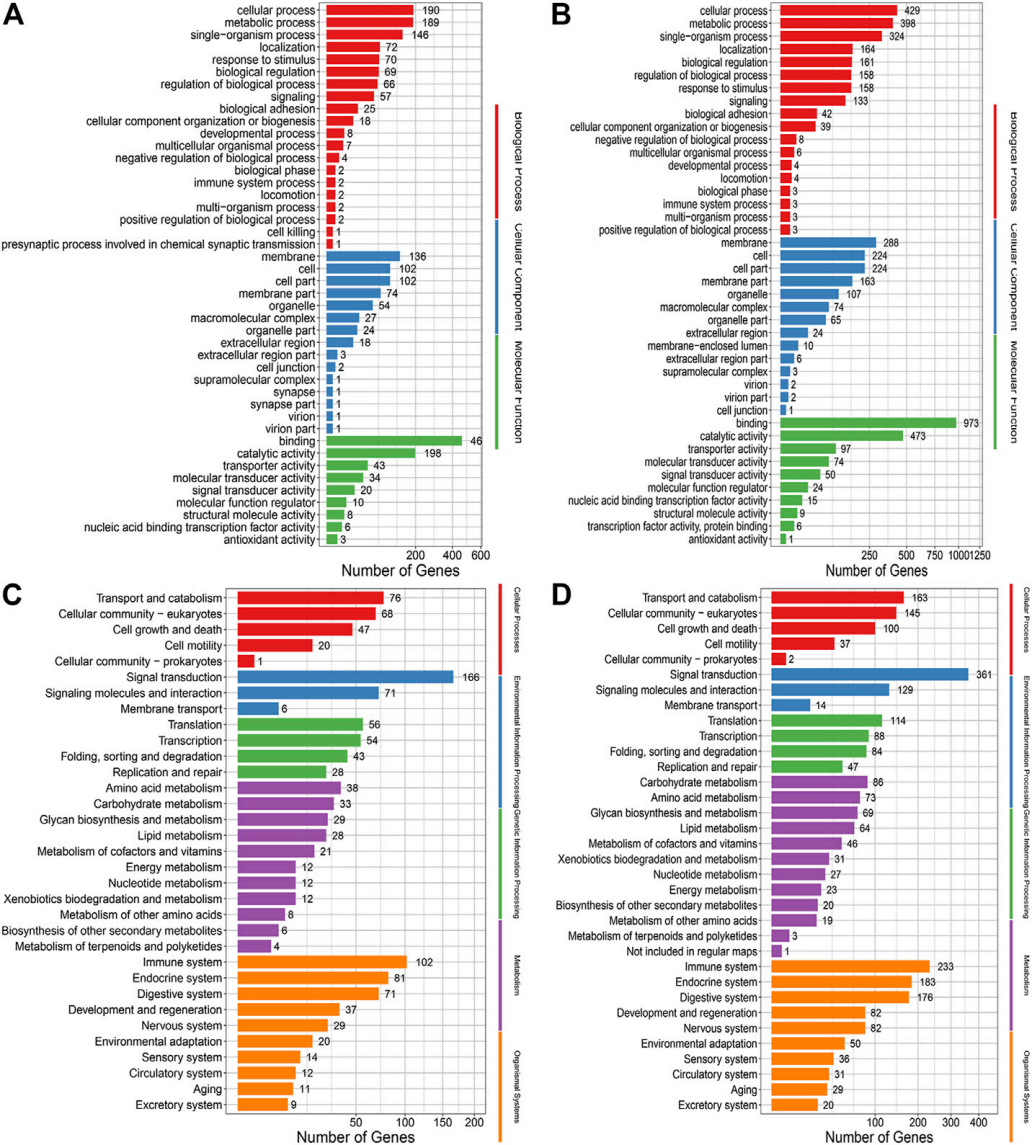
**(A)** GO annotation analysis of InDels in the control group. **(B)** GO annotation analysis of Indels in the treatment group. **(C)** KEGG enrichment analysis of Indels in the control group. **(D)** KEGG enrichment analysis of Indels in the treatment group.

Go and KEGG analysis of InDels showed that the number of genes enriched in the treated group was almost twice that of the control group. And mutations were evenly distributed across nearly all biological processes, cellular components and molecular functions, and nearly all pathways (Figure 6).

### Mutation Rates Induced by EMS Treatment

In this study, statistics were performed on data after removing mutations shared between control and treated groups. we have found that the average mutation rate as revealed from SNPs of the control group was 0.00115 ± 0.00015%, while that of the EMS treatment group was 0.01144 ± 0.00222%. And the average mutation rate as revealed from InDels of the control group was 0.000365 ± 0.000064%, while that of the EMS treatment group was 0.002281 ± 0.000473%. Taken together, the average mutation rate in the genome of the EMS treatment group (0.013721 ± 0.001347%) was much higher than that in the control group (0.001518 ± 0.000215%) (*p < 0.05*). Our results showed that the average mutation rate of the treatment group was about 9 times that of the control group.

We also found that the mutations in the treatment group tend to occur in non-coding or intronic regions, resulting in synonymous mutations or mutations with fewer Stopgain/Stoploss. The percentage of exonic SNPs in the total SNPs in the EMS treatment group (3.47 ± 0.12%) was significantly lower than that of the control group (3.63 ± 0.13%; *p* < 0.05) while the percentage of intronic SNPs in the total SNPs was not different between them (*p* > 0.05) (Table 5). The percentage of non-synonymous SNPs in the EMS treatment group (1.49 ± 0.06%) was significantly lower than that of the control group (1.83 ± 0.10%) while the percentage of synonymous SNPs in the EMS treatment group (1.93 ± 0.06%) was significantly higher than that of the control group (1.73 ± 0.03%) (*p* < 0.001 for both comparisons) (Table 6). Similarly, the percentage of Stopgain/Stoploss SNPs in the treatment group (0.032 ± 0.002%) was also significantly lower than that of the control group (0.048 ± 0.006%) (*p* < 0.001; Table 7). The percentage of InDels in the coding domains out of the total InDels in the whole genome in the treatment group (0.81 ± 0.16%) was much lower than that of the control group (1.52 ± 0.15%) (*p* < 0.001; Table 8).

### Mutations in Growth-Related Genes

To find the potential mutations that may be involved in growth regulation, we attempted to screen the mutations in growth-related genes that existed in only the large-sized animals or small-sized animals but not in the control group that were not exposed to EMS or normal-sized animals that were treated with EMS. We made a matrix using the information of nonsynonymous SNPs by first filtering for genes that contained only nonsynonymous SNP sites in the treated group and then further filtering for genes that contained only nonsynonymous SNP sites in the large individual group as well as only in the small individual group. Our results showed that mutations in major facilitator superfamily (MFS) and Tubulin were found only in the large-sized group while mutations in Homeobox and Socs were found only in the small-sized group.

## Discussion

### Effects of EMS Treatment on Hatching and Larval Growth

In this study, we found that EMS had inhibitory effects on hatching and larval growth. Similar results have also been observed in previous studies (Hou et al., 2019). We speculate that the mutation induced by EMS may affect the transcription of mRNA and disorder the expression of protein, consistent with the principle of EMS mutagenesis.

### Feasibility of Using EMS as a Mutagen in Scallops

As naturally occurring mutations are often limited for genetic breeding programs, artificial mutation induction has become a potent method for the development of novel germplasm (Penna et al., 2012). In this study, we examined the feasibility of using EMS as a mutagen in scallops breeding. We demonstrated that by treating fertilized eggs at 20 mM EMS for 3 h, we were able to induce 9 times more mutations than naturally occurring mutations. Of these mutations, about 3.47% occurred in the coding region and 1.48% were nonsynonymous mutations. These mutations may cause alteration in amino acid sequences in genes that are related to important traits and thus provide rich variations for subsequent selective breeding. Since only the mutations in the gonads may be inherited to offspring, screening of mutated individuals with remarkable phenotypes (or expected traits) in the next generation is thus needed for breeding. The mutated individuals may also provide new opportunities for the exploration of the genes related to the mutated traits. This is demonstrated by the subsequent analyses of growth-related genes utilizing the largest and smallest individuals in the treatment group, as discussed later.

### Features of EMS-Induced Mutations in Scallops

In this study, EMS induced extensive variations in the genome, with a high mutation rate of 0.014 ± 0.001%. Mutations in the format of both SNPs and InDels were induced in the mutated individuals. SNPs accounted for the majority of mutations, indicating that point mutations were the main type of variation induced by EMS treatment. Similar results have been found in *Oryzias latipes* induced by ENU (N-ethyl-N-nitrosourea), another commonly used chemical mutagen (Yoshihito et al., 2006).

After removing the shared SNPs of the controls, we found that the Ts/Tv values of both the treatment group and the control group were slightly larger than 1.0, indicating more transitions than transversions. The cause for more transitions than transversions may lie in that fact that 5-methylcytosine residues are the most prone mutation sites in the genome and can be easily deaminated to form thymine. The Ts/Tv values in both the control and the treatment group were significantly higher than the theoretical value of Ts/Tv, which is 0.5, a phenomenon named conversion deviation. It was believed that the formation of conversion deviation may be a way to reduce harmful mutations due to the selection in the long-term evolutionary process (Wakeley, 1996). Therefore, we speculate that the individuals with increased Ts/Tv in the treatment group may had a better chance of survival than those with lower Ts/Tv.

Our results also showed that there were more nonsynonymous than synonymous SNPs in the control group. In the contrary, more synonymous than nonsynonymous SNPs were found in the EMS treatment group. Nonsynonymous mutations are thought to be largely deleterious due to their property of changing amino acids. For example, it has been found that the ratio of nonsynonymous mutation/synonymous mutation involved in cancer related genes was significantly lower than that of normal genes (Chu and Wei, 2019). It is thus possible that individuals with more nonsynonymous mutations eventually did not survive, resulting in a low nonsynonymous mutation/synonymous in the EMS treatment group.

Previous studies have shown that Stopgain/Stoploss SNPs may alter protein functions (Boschiero et al., 2018). Our results also showed that the numbers of Stopgain/Stoploss SNPs in the individuals treated with EMS were significantly more than those in the control group, indicating that EMS treatment may also affect protein functions by these Stopgain/Stoploss SNPs.

Besides the SNPs in coding regions, the importance of those SNPs located in the non-coding regions cannot be overlooked. In human, almost 90% of disease-related gene variants are found to be located in the non-coding region of the genome. The variations in non-coding regions can cause diseases through alterations in gene expression by changing important regulatory elements (Rojano et al., 2016). In fact, the abundance of SNPs in noncoding regions may serve as an important source of variations for breeding programs (Arabnejad et al., 2018).

### Mutation Sites in the EMS Treatment Group

Our results showed that the mutations induced by EMS treatment distributed evenly within the genome, among all the biological processes, cellular components, molecular functions as well as pathways. While EMS seemed to induce mutations randomly in the genome, the mutations in the survivors of the treatment group tend to be less lethal to the animals. More mutations occurred in the intronic or non-coding domains, resulting in fewer non-synonymous mutations that may alter the amino acid sequences of functional proteins. The reasons for this observation may be caused by DNA repair after EMS induction and higher mortality in the individuals with lethal mutations in functional proteins. Recently, Monroe et al. (2022) indicated that mutations tend to occur at sites that are less critical to the survival of plants. The organisms seemed to have developed certain mechanisms to ensure that their important domains not affected by mutations. Our results apparently also support this hypothesis.

### Mutations in the Growth-Related Genes

In this study, we found that mutations in MFS and Tubulin were only found in large-sized individuals, suggesting that these mutations in MFS and Tubulin may be the major cause for the rapid growth in these large-sized individual. Similarly, the mutations in Homeobox and Socs were only found in small-sized individuals, suggesting that these mutations may be related to the slow growth in these small-sized individuals.

The major facilitator superfamily (MFS) is a ubiquitous group of proteins involved in the transport of a wide range of compounds (Elena et al., 2006). MFS gene is a meristem regulating gene in rice development and mutation in MFS may result in additional organ growth (Ren et al., 2017). Tubulin has been reported to be involved in cell elongation in flax (Gavazzi et al., 2017). The homeobox family is a large and diverse superclass of genes, many of which may act as transcription factors that play important roles in embryogenesis, tissue differentiation and in animals (Pearson et al., 2005; Di et al., 2015; Hench et al., 2015; Fu et al., 2021) or as master regulators for developmental genes (Ramanathan et al., 2018). Homeobox gene may also be involved in DNA repair during growth (Feltes, 2019). The SOCs are key negative regulators of cytokine and growth factor signaling (Wang et al., 2019) which may exert negative effects on cytokine signaling pathways involved in immunity, growth and development (Liu et al., 2016; Tian et al., 2020). Studies have shown that Socs gene are required for cytokine signal attenuation in mammary epithelial cells and act to limit proliferation through a negative feedback mechanism (Arun et al., 2015). In human cancer cells, activation of SOCs by epigenetic modulation through histone acetylation may induce apoptosis (Sanaei et al., 2020). Despite these lines of indirect evidence, the functions of these growth-related genes have not been well studied. Therefore, further studies are certainly needed to understand how mutations in these genes affect growth in the scallops.

## Data Availability

The original contributions presented in the study are publicly available. This data can be found here: (https://submit.ncbi.nlm.nih.gov/subs/bioproject/ BioProject ID: PRJNA810888).
